# Sex-specific neural circuits of emotion regulation in the centromedial amygdala

**DOI:** 10.1038/srep23112

**Published:** 2016-03-23

**Authors:** Yan Wu, Huandong Li, Yuan Zhou, Jian Yu, Yuanchao Zhang, Ming Song, Wen Qin, Chunshui Yu, Tianzi Jiang

**Affiliations:** 1 Key Laboratory for NeuroInformation of the Ministry of Education, School of Life Science and Technology, University of Electronic Science and Technology of China, Chengdu 610054, China; 2 Brainnetome Center, Institute of Automation, Chinese Academy of Sciences, Beijing 100190, China; 3 National Laboratory of Pattern Recognition, Institute of Automation, Chinese Academy of Sciences, Beijing 100190, China; 4 Key Laboratory of Behavioral Science, Institute of Psychology, Chinese Academy of Sciences, Beijing, 100101, China; 5 Department of Radiology, Tianjin Medical University General Hospital, Tianjin 300052, China; 6 CAS Center for Excellence in Brain Science, Institute of Automation, Chinese Academy of Sciences, Beijing 100190, China; 7 Queensland Brain Institute, The University of Queensland, Brisbane, QLD 4072, Australia

## Abstract

Sex-related differences in emotion regulation (ER) in the frequency power distribution within the human amygdala, a brain region involved in emotion processing, have been reported. However, how sex differences in ER are manifested in the brain networks which are seeded on the amygdala subregions is unclear. The goal of this study was to investigate this issue from a brain network perspective. Utilizing resting-state functional connectivity (RSFC) analysis, we found that the sex-specific functional connectivity patterns associated with ER trait level were only seeded in the centromedial amygdala (CM). Women with a higher trait-level ER had a stronger negative RSFC between the right CM and the medial superior frontal gyrus (mSFG), and stronger positive RSFC between the right CM and the anterior insula (AI) and the superior temporal gyrus (STG). But men with a higher trait-level ER was associated with weaker negative RSFC of the right CM-mSFG and positive RSFCs of the right CM-left AI, right CM-right AI/STG, and right CM-left STG. These results provide evidence for the sex-related effects in ER based on CM and indicate that men and women may differ in the neural circuits associated with emotion representation and integration.

Emotion regulation (ER) refers to individual’s ability to monitor, evaluate, and modify an emotional response, enabling the effective understanding and modulation of emotions. An individual’s capacity for emotional regulation might relate to both normal and pathological variations in general well-being and social behaviour^1^. The influence of ER on emotional experience, peripheral physiology, and neural dynamics has been well established^1^,^2^,^3^, wherein numerous studies have demonstrated sex-related difference with regard to emotional perception, experience, response, and regulation^4^,^5^,^6^,^7^. In particular, sex-related differences in emotional regulation could be an important factor precluding the divergence in psychopathology observed between males and females^8^,^9^. However, the neural basis of sex differences in ER remains poorly understood. Many studies investigating the neural basis of sex differences in emotional processing have consistently reported a significant difference with regard to amygdaloidal structure and function between genders^10^,^11^,^12^. Accordingly, the amygdala demonstrates a varying pattern of neural connectivity between males and females^13^.

The amygdala is heavily involved in ER^14^,^15^. Specifically, activity in the amygdala is purposefully stimulated or inhibited by feedback from the cerebral cortex, enabling people to regulate emotions in a manner that suggests a particular sensitivity of the subjective emotional state to negative information^16^,^17^. The amygdala is a frequent focus of sex difference-based research and is typically considered a key structure in mediating the sex-based variations in emotional response observed between males and females^10^,^18^. The amygdala is a complex structure with multiple sub-regions that can be anatomically parcellated into three nuclei: the laterobasal (LB), superficial (SF), and centromedial (CM), each of which features distinct functions and a varying pattern of connectivity^19^,^20^. The LB nuclei are primarily engaged in the updating and evaluation of emotional stimuli, typically through innervations by cortical and subcortical regions, including the thalamus, hippocampus, and prefrontal cortex^15^,^21^,^22^,^23^. The CM group, composed of the central and medial nuclei, mediates behavioral responses to potentially harmful stimuli via connectivity with the brainstem, in addition to cortical and striatal regions such as the caudate nucleus^21^,^24^,^25^. An output region of the amygdala, the CM nuclei are also reported to facilitate motor responding and reward processing, while simultaneously modulating attentional allocation and cortical vigilance^20^,^24^. The SF sub region lies adjacent to the laterobasal group and includes the cortical nuclei involved in olfactory and olfactory-related processing^26^,^27^. However, the association of amygdaloidal sub-regions with sex-related differences in ER and the manifestation of regulation in neural networks remain unclear.

Resting-state functional magnetic resonance imaging (fMRI) has been used increasingly in recent years to identify enduring and intrinsic properties of the brain^28^,^29^. Resting-state functional connectivity (RSFC) can also be used to draw correlations between functional differences in neural networks and individual variations in behavioral traits, including personality and emotional intelligence^30^,^31^. Relatively less is known about the fixed neural correlates of individual differences in ER. To our knowledge, only one study has explored the neural correlates of reappraisal (an ER strategy) using RSFC^32^. Using a seed-based RSFC approach, individuals with greater reappraisal ability demonstrated reduced functional connectivity (FC) between the right amygdala and the right medial prefrontal cortex (MPFC) and posterior cingulate cortex (PCC); and reduced FC between both the left and right dorsolateral prefrontal cortical (dlPFC) and posterior visual regions. However, this study did not address the neural correlates of sex differences with regard to variations in ER trait level.

In the present study, we investigated sex-related differences in ER by examining the FC patterns of amygdaloidal sub regions using resting-state fMRI. FC patterns were then cross-referenced with variations in ER. Given the general role of the LB nuclei in the evaluation of emotion, that of the CM in modulating emotional response^20^,^27^, and considering the sex differences identified in emotional evaluation and response^33^,^34^,^35^,^36^, we hypothesized that the neural correlates of ER in males might differ from females in the LB and CM. Emotional processing in the amygdala is thought to be governed by top-down systems, mediated by regions such as the dlPFC, dorsomedial prefrontal cortex (dmPFC), and anterior cingulate (ACC). Semantic systems, such as the superior temporal gyrus (STG), have also been implicated in the modulation of emotion generation systems in the amygdala^1^,^37^,^38^. Therefore, we further speculated that variations in amygdala connectivity with regard to ER might also be replicated in top-down cortical and semantic systems.

## Results

### Behavioral results

Cronbach’s alpha for the Emotional Intelligence Scale (EIS) items in the current study was α = 0.84, indicating that the internal consistency of EIS in this dataset was good^39^. A confirmatory factor analysis (CFA) was also performed on the Chinese version of EIS. For the original four-factor 19-item structure, χ^2^/*df*, comparative fit index (CFI), goodness-of-fit index (GFI), and root mean square error of approximation (RMSEA), were found to be 1.77, 0.89, 0.87, and 0.05, respectively. The CFA showed that the 19-item four-factor structure of the EIS had a reasonable fit to the dataset and the emotion monitoring and control subscale was reliable in the current dataset.

Demographic information for the 258 healthy subjects included in the final analysis is provided in Table 1. The male participants had higher BDI scores, ER scores and mean framewise displacement (FD) values than the female group (*p* = 0.024; *p* = 0.021; *p* = 0.035); the females were older (*p* = 0.004) and higher in their STAI-S scores (*p* = 0.012) and STAI-T scores (*p* = 0.023) than the males. Pearson correlation tests found ER scores had a significant negative correlation with STAI-S scores (*r* = −0.29, *p* < 0.001), STAI-T scores (*r* = −0.31, *p* < 0.001) and BDI scores (*r* = −0.32, *p* < 0.001). No correlation was found between their ER scores and FD values (*r* = 0.002, *p* = 0.098).

### Sex-related difference in amygdala subregions connectivity

Results from whole-brain connectivity analyses using the amgdala clusters as seed regions are shown in Figure S2. Connectivity maps were largely similar for seed regions in male and female group. The two-sample t test analysis found significant sex differences only in the SF. Compared to women, greater RSFCs were found in the left inferior/middle frontal gyrus (IFG/MFG), right IFG and the right inferior parietal lobe (IPL) based on the left SF seed in males and also found the left IFG, the right IFG/STG and the left Calcarine based on the right SF seed in males (Table S1).

### ER by sex interaction based on amygdala subregions

Our data revealed that on the amygdala subregion level, the ER by sex interaction was significant for the RSFC in the right CM. Specifically, the ER by sex interaction was significant for negative RSFC between the right CM and the medial superior frontal gyrus (SFG) and for positive RSFC between the right CM and the left superior temporal gyrus (STG), the left anterior insula (AI) and the right AI (including the right STG). The independent analyses of the average RSFC values in the four regions validated the interaction effects [for the medial SFG (β = −0.038, *p* < 0.001), for the left AI (β = 0.033, *p* < 0.001), for the right AI/STG (β = 0.043, *p* < 0.001), and for the left STG (β = 0.038, *p* < 0.001)].

**Figure 1:**
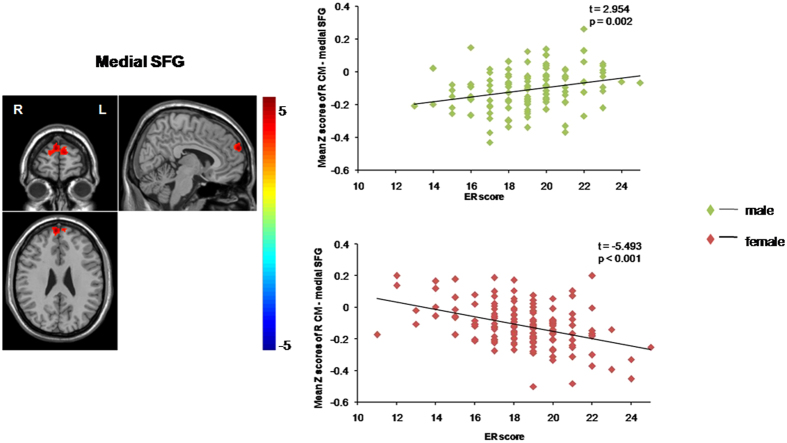
Functional connectivity pattern between the right CM and the medial SFG and the correlation with ER scores. Scatter plots showing ER-related changes in RSFC in the male and female groups, separately. The lines are linear fits of the data. Abbreviations: CM, centromedial subregion; medial SFG, medial superior frontal gyrus; ER: emotion regulation; RSFC, resting-state functional connectivity; L, left; R, right.

**Figure 2:**
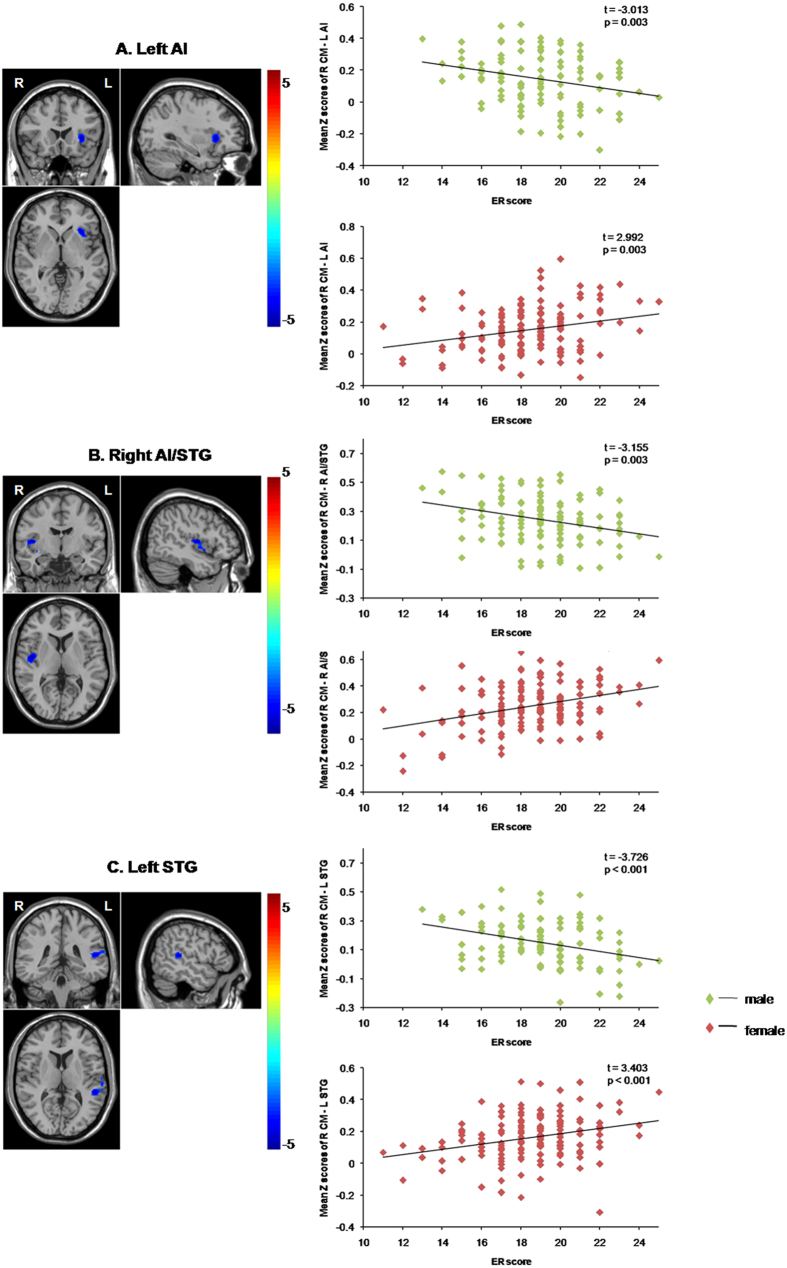
Functional connectivity patterns between the right CM and the left AI, the right AI/STG and the left STG and their correlations with ER scores. (**A**) The left AI, (**B**) The right AI/STG and (**C**) the left STG. Scatter plots showing ER-related changes in RSFC in the male and female groups, separately. The lines are linear fits of the data. Abbreviations: CM, centromedial subregion; AI, anterior insula; STG: superior temporal gyrus; ER, emotion regulation; RSFC, resting-state functional connectivity; L, left; R, right.

Post-hoc tests of these effects showed that the negative RSFC in the medial SFG was positively correlated with the ER scores for the males (β = 0.015, SE = 0.005, *t* = 2.954, *p* = 0.003), but negatively correlated with the ER scores for the females (β = −0.023, SE = 0.004, *t* = −5.493, *p* < 0.001), suggesting that a stronger negative RSFC between the medial SFG and the right CM, predicted a stronger trait-level ER in the females and a weaker trait-level ER in the males. The positive RSFC in the three regions, the left AI, the right AI/STG and the left STG, were positively correlated with the ER scores for the females (β = 0.015, SE = 0.005, *t* = 2.992, *p* = 0.003; β = 0.023, SE = 0.005, *t* = 4.288, *p* < 0.001; β = 0.017, SE = 0.005, *t* = 3.403, *p* < 0.001, respectively), but negatively correlated with the ER scores for the males (β = −0.018, SE = 0.006, *t* = −3.017, *p* = 0.003; β = −0.020, SE = 0.006, *t* = −3.155, *p* = 0.003; β = −0.021, SE = 0.006, *t* = −3.726, *p* < 0.001, respectively), suggesting that a stronger positive RSFC between the right CM and the left AI, the right PI/STG and the left STG, predicted a stronger trait-level ER in the females and a weaker trait-level ER in the males. (Figs 1 and 2, Table 2).

## Discussion

Using RSFC, the present study examined the sex-related differences between neural correlates of ER in amygdaloidal sub-regions. Initially, RSFC in the SF nucleus was found to be stronger in males compared with females. Moreover, the neural correlates of trait-level ER differed significantly between males and females, particularly within resting-state functional networks in the CM nucleus. The study also identified sex-related variations in the functional activity of the medial SFG, left AI, right AI/STG, and left STG regions that are reportedly recruited in ER tasks^40^,^41^,^42^. In particular, females who demonstrated enhanced trait-level ER featured enhanced RSFC with the CM, whereas males who demonstrated stronger trait-level ER displayed weaker RSFC with the CM nucleus. Such results indicate that CM FC patterns display sex-specific variations in their association with trait-level ER.

The present study examined the extent of sex difference in the RSFC of amygdaloidal sub-regions and identified significant variations between sexes in the RSFC of the SF nucleus. A recent study, which reported similar sex-related differences in RSFC in the SF nucleus^43^, supported these findings. In the present study, males demonstrated a stronger RSFC in the IFG and the right IPL based on the left SF, and in the IFG and the left calcarine based on the right SF. Evidence suggests that the SF nucleus supports olfactory information processing and olfaction-related affective processing in rodents, underlying the observations made in the present study, where in the SF nucleus positively predicted activity throughout the limbic cortex^20^. Areas of functional coupling areas within the SF nucleus were reported to incorporate cognitive and sensorimotor function, for example, the IFG for cognitive control function^44^,^45^, and the IPL for sensory processing and sensorimotor integration^46^. These findings were partially consistent with the previous literature, where in males demonstrated stronger FC within sensorimotor networks^46^,^47^,^48^ and between cognitive and sensory networks^48^. With increasing age, males also displayed stronger RSFC between the SF nucleus and parieto-occipital cortex^43^. The stronger cognitive functional coupling observed within the SF in males suggests that resting-state functional network analysis can provide a reliable measure for the neural correlates of trait-level ER and account for functional variations linked to sex difference.

Importantly, the sex differences associated with the ER trait-level was only observed in the CM. This may suggest that sex-related difference of ER trait level exist only in a specific sub-region of the amygdala. This is supported by previous studies that indicated that the LB for the perception and generation of emotion processing and the CM for controlling automatic expressions of emotion^20^,^49^. In particular, the LB is the main nucleus that receives sensory inputs from sensory systems^27^ and is thought to play a pivotal role in evaluating emotional and sensory stimuli^50^. So the LB can be viewed as an “input” amygdala complex for emotion generation and evaluation. Just as the LB is the sensory gateway into the amygdala, the CM is believed to be an important output region^49^. In fact, the CM receives convergent information from several other amygdaloid and cortical regions and sends efferent extensions to various subcortical structures for the expression of innate emotional responses and their associated physiological responses^49^. More specifically, the medial nucleus of the amygdala has long been known to be sexually dimorphic^51^ and reflects sex differences^52^. Therefore, the CM may be an important “output” amygdala subregion that mediates affective-related behavioral responses. In brief, the sex differences that are related to the correlation between connectivity and ER in the CM may suggest sex-specific patterns that primarily occur in the “output” of emotion in the CM rather than the “input” of emotion in the LB.

Significant sex-related differences associated with ER in the medial SFG, left AI, right AI/STG, and left STG, were identified in the present study, all of which are reportedly recruited in emotional regulation tasks^40^,^41^,^42^. Recently, Ochsner, *et al*.^38^ proposed a model of ER that specified the role of systems such as the prefrontal and cingulate areas in the modulation of activity in affective, perceptual, and semantic systems. Consistent with this model of ER, the present study identified sex-related differences in functional activity in the medial SFG (an area implicated in emotion regulation), the insula (an area involved in affective/emotion generation and representation), and the STG (a region implicated in perceptual and semantic processing)^38^.

The medial SFG plays a critical role in emotional regulation, appraisal and response^17^,^53^,^54^, and has been implicated in the neural representation of internal states^55^. In the present study, activation in the medial SFG demonstrated an inverse correlation with that of the right CM. In previous studies, activity of the medial SFG demonstrated a negative correlation with the functional activity of the amygdala in response to negative scenes^56^,^57^. This suggests that the extent of coupling between the medial SFG and amygdala is related to effective ER^14^,^58^.

The insula features a general role in emotional processing. Especially, the AI demonstrates involvement in interoceptive awareness^38^,^59^. In addition, the AI was activated during interpersonal emotional regulation^42^. Moreover, a recent emotional regulation study also implicated AI-amygdala coupling, where in an increase in the activation of the amygdala and the AI was accompanied by a decrease in negative emotional experience after suppression^60^. This coupling is consistent with the positive functional correlation observed between the AI and the CM nucleus in the present study.

An integral component of the perceptual and semantic systems, the STG similarly demonstrated a positive correlation with the CM nucleus, featuring enhanced connectivity during emotional regulation. In previous studies, the STG featured reciprocal connections with the amygdala during social perception tasks^61^ and demonstrated increased activity in conjunction with the amygdala during emotional processing^62^. Such findings might partially support the positive correlation identified in the present study between STG and amygdala function. In particular, increasing evidence exists to suggest that the STG is involved in emotional processing and social perception^63^, especially the representation of emotional information during the initial stages of emotional regulation^40^,^64^. In brief, the prevalence of sex defference in ER in the medial SFG, insula, and STG indicates that males and females might feature a functional divergence in the neural circuits associated with emotional generation, representation, and integration.

Specifically in females, a higher trait-level of ER was associated with increasingly negative RSFC between the right CM nucleus and the medial SFG. In addition, a higher level of ER correlated with increasingly positive RSFC between the right CM nucleus and the left AI, right AI/STG, and left STG. Such findings are supported by previous task-based fMRI studies, in which increased activity was identified in the medial SFG and decreased activity was found within the amygdala after effective ER in females but not males^65^. Furthermore, the activity of both the insula and the amygdala was increased after successful ER (using the suppression strategy) in females^60^. Accordingly, during periods of stress, the STG was more strongly activated in females than males^66^ and was found to indirectly influence emotional responses in the amygdala^67^. Therefore, it is reasonable to suggest that the stronger coupling identified between the right CM and the medial SFG, left AI, right AI/STG, and the left STG predicted a higher trait-level of ER within females in the current study. The above finding might provide support for the notion that females with a higher trait-level of ER feature a greater level of internal- and emotional-focus, compared to males. In particular, females reportedly demonstrate a greater tendency to focus on emotional experience, in addition to acknowledging and discussing emotional behaviour more openly than males^68^. They are also more likely to apply emotion-focused strategies^33^, which recruit an increasing number of regions associated with emotion, including the insula, amygdala, orbital frontal cortex, and the medial prefrontal cortex^5^,^69^. Additionally, the medial SFG, insula, and STG play important roles in general emotional processing and the representation of internal states^70^. The medial SFG receives direct inputs regarding the internal state of the body from the insula and STG and might be important for the regulation of internally-focused mechanisms^64^.

However, similar patterns of connectivity were associated with diverging levels of ER in males and females. In males, decreased negative RSFC observed between the right CM and medial SFG, and positive RSFC observed between the right CM-left AI, right CM-right AI/STG, and right CM-left STG were associated with a higher trait-level of ER. McRae, *et al*.^65^ found that, despite comparable decreases in self-reported negative emotion in males and in females during an effort-based ER task (requiring cognitive reappraisal to down-regulate negative emotion), males demonstrated a smaller increase in prefrontal activation and a greater decrease in amygdala activation than females. The findings of the present study indicate that males with higher levels of ER may be more effective at ER than females. In the current study, males demonstrated higher overall scores of ER, indicating a higher trait-level of ER than females. This is consistent with previous findings that males possess a greater ability to regulate their emotions than females^71^.

Emotional dysregulation is becoming increasingly viewed as central to several types of psychopathology^72^. Although the present study did not directly examine emotional dysregulation, an association was noted between lower ER scores and a reduced capacity for ER. ER scores demonstrated a negative correlation with STAI-S scores, STAI-T scores, and BDI scores. This is congruent with existing literature, which documents that poor ER is associated with a number of clinical states, including depression and anxiety^73^. At rest, in the current study, a lower level of ER was associated with decreased RSFC between the right CM and several regions (medial SFG/insula/STG) that have been associated with emotion in females, compared to males. Accordingly, this data partially supports the notion that a decrease in RSFC in females might be related to certain types of psychopathology. Since ER deficits also feature at the core of several psychopathologies, which also feature diverging prevalence and presentation based on the effects of gender^9^, further work with regard to sex difference in the neural circuitry of ER will be important.

Two limitations of this study can be noted. Firstly, the current study did not assess the specific strategies that males or females prefer to use for ER. Males and females might prefer to use different ER strategies, wherein males tend to apply more cognitive-focused strategies and females tend to use more emotion-focused strategies^4^. This divergence in ER strategies might underlie the differences observed in FC patterns associated with ER. Secondly, no increase in the coupling of key cognition-related areas, such as the lateral prefrontal cortex (LPFC) or the ACC, was associated with the level of ER in males. In the present study, however, the FC associated with sex differences was examined based only on the amygdala, an area known to be part of the emotion generation system^38^. The involvement of cognition-related areas such as the LPFC and ACC might have been observed if the study had been based on other regions of the emotion generation system, such as the insula and posterior visual cortex. For example, greater functional coupling of the ACC with the insula was identified more frequently in males than females when experiencing affection^5^.

In conclusion, the present study demonstrated sex-specific FC patterns of the CM nucleus, which were associated with the level of ER in a group of healthy Chinese subjects. The sex differences in ER with regard to the cortico/subcortico-limbic circuits between the CM nucleus and the insula/STG/medial SFG indicate that males and females might demonstrate functional differences in the neural circuits associated with emotional representation and integration. These findings might provide a better understanding of the mechanisms underlying sex difference with regard to ER.

## Materials and methods

### Subjects

Three hundred and twenty-three young, right-handed subjects were enrolled in this study. Careful screening was performed to ensure that all the volunteers were native Chinese speakers with no history of psychiatric or neurological illness, psychiatric treatment, or drug or alcohol abuse, and without MR contraindications. We also used the Spielberger State-Trait Anxiety Inventory (STAI)^74^ and the Beck Depression Inventory-II (BDI-II)^75^ to measure anxiety and depression for each individual. To ensure that our sample population represented a normally functioning subjects, only volunteers with BDI <20 and STAI-trait <54 and STAI-state <54 were included. Then we excluded 15 participants who had high anxiety or depression scores. 11 participants were excluded due to errors in the raw imaging data after two board-certified radiologists, Yu and Qin, manually inspected the raw fMRI data; an additional 28 participants were excluded due to a lack of behavioral data, or extreme ER scores (in excess of three standard deviations from the mean; ER ≤10, only one participant). Finally, we excluded an additional 11 subjects who had excessive head movement. This left 113 men (mean age = 22.27 years, SD = 2.52 years) and 145 women (mean age = 23.12 years, SD = 2.18 years) for a total of 258 subjects in the final group. The study was approved by the local Research Ethics Committee of Tianjin Medical University. All the experiments and the methods were performed in accordance with the experimental guidelines of the Tianjin Medical University and all subjects signed an informed-consent before his/her participation.

### ER measurement

We used the Emotional Monitoring and Control subscale from EIS Chinese Revised Version to test each subject’s trait-level ER^76^. The Chinese revised version of the EIS is a self-report measure of emotionally and socially intelligent behavior, which provides an estimate of an individual’s underlying emotional and social intelligence^76^. The EIS consists of 19 items and four scaled factors, which are emotion evaluation, emotion utilization, social ability, and emotion monitoring and control. The emotional monitoring and control factor, which measures how individuals monitor and control their feelings, contains five items, which indicate the extent, primarily at the trait-level, to which an individual can effectively regulate his/her emotions. Items are randomized to avoid a response bias. The current study used the emotional monitoring and control section of the Chinese version and referred to the results as an individual’s ER score. The factor scales consist of self-descriptive statements scored on a five-point scale ranging from Strongly Disagree to Strongly Agree, giving a range of total scores between 0 and 25 for each quotient. Lower ER scores indicated the lower trait-level ER and less successful ER, and higher ER scores indicated the higher trait-level ER and more successful ER.

### MRI data acquisition

MR images were acquired on a 3.0 Tesla MR scanner (General Electric, Milwaukee, WI, USA). During scanning, foam pads and earplugs were used to limit head motion and to reduce scanning noise respectively. All participants received a high resolution T1-weighted brain volume (BRAVO) 3D MRI sequence for obtaining T1 images (repetition time (TR) = 8.1 ms, echo time (TE) = 3.1 ms, 176 sagittal slices, flip angle = 13°, voxel size = 1 mm × 1 mm × 1 mm). After structural imaging, functional imaging during resting-state took place with a single-shot, gradient-echo, echo-planar-imaging (SS-GRE-EPI) sequence sensitive to BOLD contrast. The parameters for resting-state fMRI were as follows: 40 slices, 180 volumes, TR = 2000 ms, TE = 30 ms, no gap, voxel size = 3.75 mm × 3.75 mm × 4.0 mm, FOV = 240 × 240 mm, matrix = 64 × 64, flip angle = 90°. During resting-state fMRI, all the subjects were instructed to lie still with their eyes closed, to move as little as possible, to think about nothing in particular, and not to fall asleep.

### Data preprocessing

Data processing was performed using the Data Processing Assistant for Resting-State fMRI Toolbox (DPARSF, http://www.restfmri.net/forum/DPARSF)^79^. The following preprocessing steps were performed: discarding the first 10 slices, slice-timing correction, head motion correction, spatial normalization, resampling to 2 × 2 × 2 mm^3^, smoothing with a 4 mm full-width at half-maximum Gaussian kernel, regressing nuisance signals (6 motion parameters, global mean signal, and average blood oxygen level-dependent signals in ventricular and white matter regions), and temporal filtering (0.01–0.1 Hz). The participants who had a maximum displacement greater than 2 mm in any of the cardinal directions (x, y, z) or a maximum spin (x, y, z) greater than 2° were excluded from subsequent analysis.

### Framewise displacement (FD)

This measure indexes the movement of the head from one images to the next, and is calculated as the empirical sum of the rigid-body motion between consecutive volumes in all directions^77^,^78^. We used the following formula for FD, 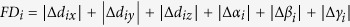, where 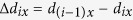, and similarly for the other head realignment parameters [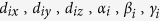]. 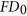 is set to zero by convention. Rotational displacements were converted from degrees to millimeters by calculating displacement on the surface of a sphere of radius 50 mm^78^.

### Regions of interest

The bilateral amygdala sub-regions were determined using stereotaxic, probabilistic maps of cytoarchitectonic boundaries developed by Amunts, *et al*.^19^ and implemented in FSL’s Juelich histological atlas. In order to avoid the potential artifact caused by smoothing and registration error, we created our regions of interest (ROIs) using a 50% probabilistic map. The superficial (SF) sub-region consists of the anterior amygdaloid area, the amygdalopyriform transition area, the amygdaloid-hippocampal area, and the ventral and posterior cortical nuclei. The centromedial (CM) sub-region includes the central and medial nuclei. The laterobasal (LB) sub-region consists of the lateral, basolateral, basomedial, and paralaminar nuclei. A similar division of the human amygdala has been used in several previous studies^20^,^80^. The six amygdala seeds are seen in the Figure S1 and the voxel size of each seeds is respectively 233 (lest LB), 244 (right LB), 131 (left SF), 100 (right SF), 26 (left CM) and 32 (right CM).

### Resting state functional connectivity networks

For the left and right LB, CM and SF seed, we calculated correlation coefficients between each seed time series and all other voxels time series, resulting in a correlation map for each subject. Then a Fisher r-to-z transformation was used to transform the correlation coefficient to Z values to improve normality. Finally, individual *z*-score RSFC maps were obtained for further analysis.

### Statistical analysis

First, ER scores for a large sample were evaluated using the Emotional Monitoring and Control subscales from the Chinese version of the EIS. The EIS features a four-factor structure, and has been previously validated using Chinese samples^76^. In order to confirm that the four-factor model would fit data obtained in the present study, a CFA was applied, utilizing Amos 4.0. The CFA is typically used to determine whether observed data fits an existing model, specifically, in this incidence, the four-factor model that was proposed by Huang, *et al*.^76^.

Next, a one-way ANOVA was applied to assess for significant differences between the means of both genders with regard to the screening variables of age, BDI score, STAI-S score, STAI-T score, and mean FD value using SPSS version 20.0 (SPSS Inc., Chicago, IL, USA). Screened variables were introduced as covariates in the statistical analysis of sex difference using a general linear model (GLM).

Furthermore, for the left and right LB, CM, and SF nuclei, a one-sample t test was carried out for the *z*-score RSFC maps of male and female participants using family-wise-error (FWE) rate correction. The significance threshold was set at *p* < 0.05. To investigate sex-related differences between the FC of each seed, a two-sample t test was applied to compare the resulting *z*-score RSFC maps between male and female groups. As before, significance was set at a threshold of *p* < 0.05, and FWE rate correction was reported. To examine the effects of sex on the relationship between ER scores and FC patterns (positive and negative) of the left and right LB, CM and SF, a random-effect full factorial model was utilized. In this context, gender was assigned as a factor and ER scores as an interaction covariate. Age, mean FD values, BDI, STAI-S, and STAI-T scores were regressed as covariates. A cerebral cortex explicit mask was applied. Cluster-level FWE correction was performed throughout the whole brain, and a two-stage procedure was followed: (1) the image underwent thresholding using a voxel-wise threshold of *p* < 0.001 (uncorrected); (2) Cluster-level inference was then used to retain clusters that were large enough to surpass the given threshold (family-wise) of *p* < 0.05. All FC analyses were performed using Statistical Parametric Mapping 8 (SPM8)^81^, wherein the age, mean FD values, depression and anxiety scores of each subject were regressed.

To investigate the significance of sex × ER interaction effects detected using SPM8, the connectivity correlation coefficient (z value) was extracted and averaged over the voxels within each significant cluster for both gender groups. Sex × ER interaction effects were then analysed for the z value and ER scores using SPSS v20.0. If an interaction effect was identified as statistically significant, post hoc tests were then performed using the MODPROBE tool. This was used to probe single-degree-of-freedom interactions for ordinary least squares and logistic regression analyses^82^ in SPSS.

Finally, to examine the existence of an association between ER scores and the altered FC observed in amygdaloidal sub-regions, correlation analyses were performed using SPSS. The significant threshold value was set at *p* < 0.05.

## Additional Information

**How to cite this article**: Wu, Y. *et al*. Sex-specific neural circuits of emotion regulation in the centromedial amygdala. *Sci. Rep*. **6**, 23112; doi: 10.1038/srep23112 (2016).

## Supplementary Material

Supplementary Information

## Figures and Tables

**Table 1:** Subject demographics by sex.

Measure	Men (N = 113)			Women (N = 145)			p-value
	Mean	SD	Range	Mean	SD	Range	
Age	22.27	2.52	18–29	23.12	2.18	18–29	0.004
ER scores	19.04	2.33	13–25	18.44	2.42	11–25	0.021
BDI scores	7.79	5.56	0–19	6.25	5.2	0–19	0.024
STAI-State	30.64	6.39	20–52	32.72	6.72	20–50	0.012
STAI-Trait	34.88	7.02	20–53	36.88	7.03	20–54	0.025
FD value	0.08	0.04	0.04–0.29	0.08	0.03	0.03–0.24	0.035

Abbreviations: ER scores, Emotion Regulation scores; BDI scores, Beck Depression Inventory scores; STAI-State, State-Trait Anxiety Inventory-State; STAI-Trait, State-Trait Anxiety Inventory-Trait; SD, standard deviation.

**Table 2:** Sex by the ER score interaction effects on the RSFC based on the right CM.

Brain regions	Clustersize	BA	MNIcoordinates	PeakF-value
L/R medial SFG	364	10	6 62 26	4.82
L STG	229	22	−52 −38 10	−4.42
R AI/STG	216	13/22	46 −8 6	−4.63
L AI	158	13	−32 20 0	−4.55

Abbreviations: RSFC, resting-state functional connectivity; CM, centromedial subregion; L, left; R, right; medial SFG: medial superior frontal gyrus; STG: superior temporal gyrus; and AI, anterior insula.
