# Supplementary material for: Sex-specific neural circuits of emotion regulation in the centromedial amygdala
Source: Sci Rep. 2016 Mar 23;6:23112. doi: 10.1038/srep23112 (PMC4804331; doi:10.1038/srep23112)
Supplement: Supplementary Information [file srep23112-s1.doc]

# Sex-specific neural circuits of emotion regulation in the centromedial amygdala

## Running title: Sex-specific patterns of emotion regulation

Yan Wu^1, #^, Huandong Li^2, 3,#^, Yuan Zhou^4^, Jian Yu^1^, Yuanchao Zhang^1^, Ming Song^2, 3^, Wen Qin^5^, Chunshui Yu^5^, Tianzi Jiang^1,2,3,6,7,*^

^1^Key Laboratory for NeuroInformation of the Ministry of Education, School of Life Science and Technology, University of Electronic Science and Technology of China, Chengdu 610054, China

^2^Brainnetome Center, Institute of Automation, Chinese Academy of Sciences, Beijing 100190, China

^3^National Laboratory of Pattern Recognition, Institute of Automation, Chinese Academy of Sciences, Beijing 100190, China

^4^ Key Laboratory of Behavioral Science, Institute of Psychology, Chinese Academy of Sciences, Beijing, 100101, China

^5^Department of Radiology, Tianjin Medical University General Hospital, Tianjin 300052, China

^6^CAS Center for Excellence in Brain Science, Institute of Automation, Chinese Academy of Sciences, Beijing 100190, China

^7^The Queensland Brain Institute, The University of Queensland, Brisbane, QLD 4072, Australia

^*^ **Corresponding author**:

Tianzi Jiang, Brainnetome Center, Institute of Automation, Chinese Academy of Sciences, Beijing 100190, China

Phone: +86-10-8254 4778; Fax: +86-10-8254 4777 Email: [jiangtz@nlpr.ia.ac.cn](mailto:jiangtz@nlpr.ia.ac.cn)

## Outline

**Sex-specific neural circuits of emotion regulation in the centromedial amygdala**

……………………………………………………………………………………….1

The Chinese revised version of the emotional intelligence scale ……………………………..…3

Figure S1. The location of each amygdala subregion…………………………………………….5

Figure S2. The patterns of RSFC for each amygdala subregion in the males and females, separately

…………………………………………………………………………………………………….6

Table S1. Sex effects based on left and right SF ……………………………………………7

### The Chinese revised version of the emotional intelligence scale

The Chinese revised version of the EIS is a self-report measure of emotionally and socially intelligent behavior, which provides an estimate of an individual’s underlying emotional and social intelligence . The Emotional Intelligence Scale consists of 19 items and four scaled factors, which are emotion evaluation, emotion utilization, social ability, and emotion monitoring and control.

The emotional monitoring and control factor, which measures how individuals monitor and control their feelings, contains five items, which indicate the extent, primarily at the trait-level, to which an individual can effectively regulate his/her emotions. Lower ER scores indicated less successful emotion regulation, and higher ER scores indicated more successful emotion regulation.

Cronbach’s alpha for the EIS items in the current study was α = 0.84, indicating that the internal consistency of EIS in this dataset was good . A CFA was also performed on the Chinese version of EIS. For the original four-factor 19-item structure, χ^2^/*df*, comparative fit index (CFI), goodness-of-fit index (GFI), and root mean square error of approximation (RMSEA), were found to be 1.778, 0.89, 0.87, and 0.055, respectively. The CFA showed that the 19-item four-factor structure of the EIS had a reasonable fit to the dataset and the emotion monitoring and control subscale was reliable in the current dataset.

**The emotion monitoring and control subscale**

1. I have control over my emotions (emotional control).
2. I know when to speak about my personal problems to others (emotional control).
3. I know why my emotions change (emotional monitoring).
4. When I experience a positive emotion, I know how to make it last (emotional control).
5. When another person tells me about an important event in his or her life, I can show as though I have experienced this event myself (emotional control).

**The emotion utilization subscale**

1. Some of the major events of my life have led me to re-evaluate what is important and not important.
2. I am aware of my emotions as I experience them.
3. Emotions are one of the things that make my life worth living.
4. When my mood changes, I see new possibilities.
5. When I am in a positive mood, I am able to come up with new ideas.

**The social ability subscale**

1. I help other people feel better when they are down.
2. I present myself in a way that makes a good impression on others.
3. I seek out activities that make me happy.
4. Other people find it easy to confide in me.
5. I expect that I will do well on most things I try.

**The emotion evaluation subscale**

1. I am aware of the non-verbal messages I send to others.
2. By looking at their facial expressions, I recognize the emotions people are experiencing.
3. I know what other people are feeling just by looking at them.
4. It is difficult for me to understand why people feel the way they do.

**References**

Cronbach, L. (1951). Coefficient alpha and the internal structure of tests. *Psychometrika, 16*, 297-334.

Huang, Y. H., Lu, A. Q., Wang, L., & Shi, J. Q. (2008). Validation of the emotional intelligence scale. *Acta Sci Natur Univ pekinensis, 44*, 970-976.

## Figure S1. The location of each amygdala subregion.


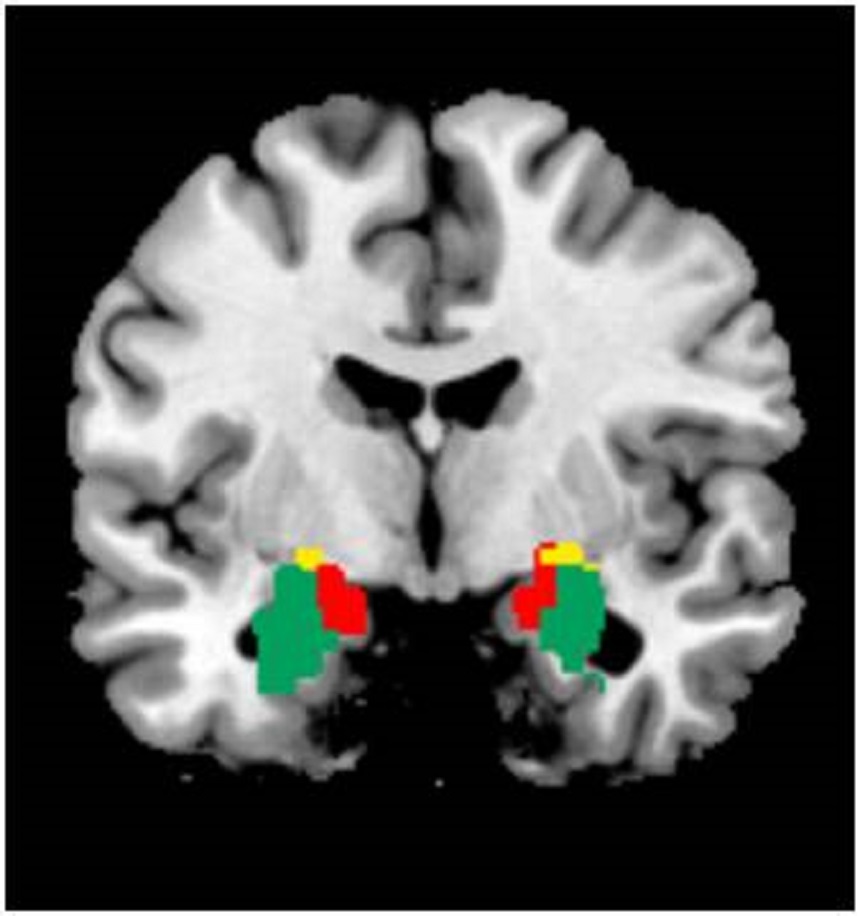


Figure S1. The location of each amygdala subregion (green = LB; yellow = CM; and red = SF). Abbreviations: CM, centromedial subregion; L, left; LB, laterobasal subregion; R, right; and SF, superficial subregion.

## Figure S2. The patterns of RSFC for each amygdala subregion in the males and females, separately.


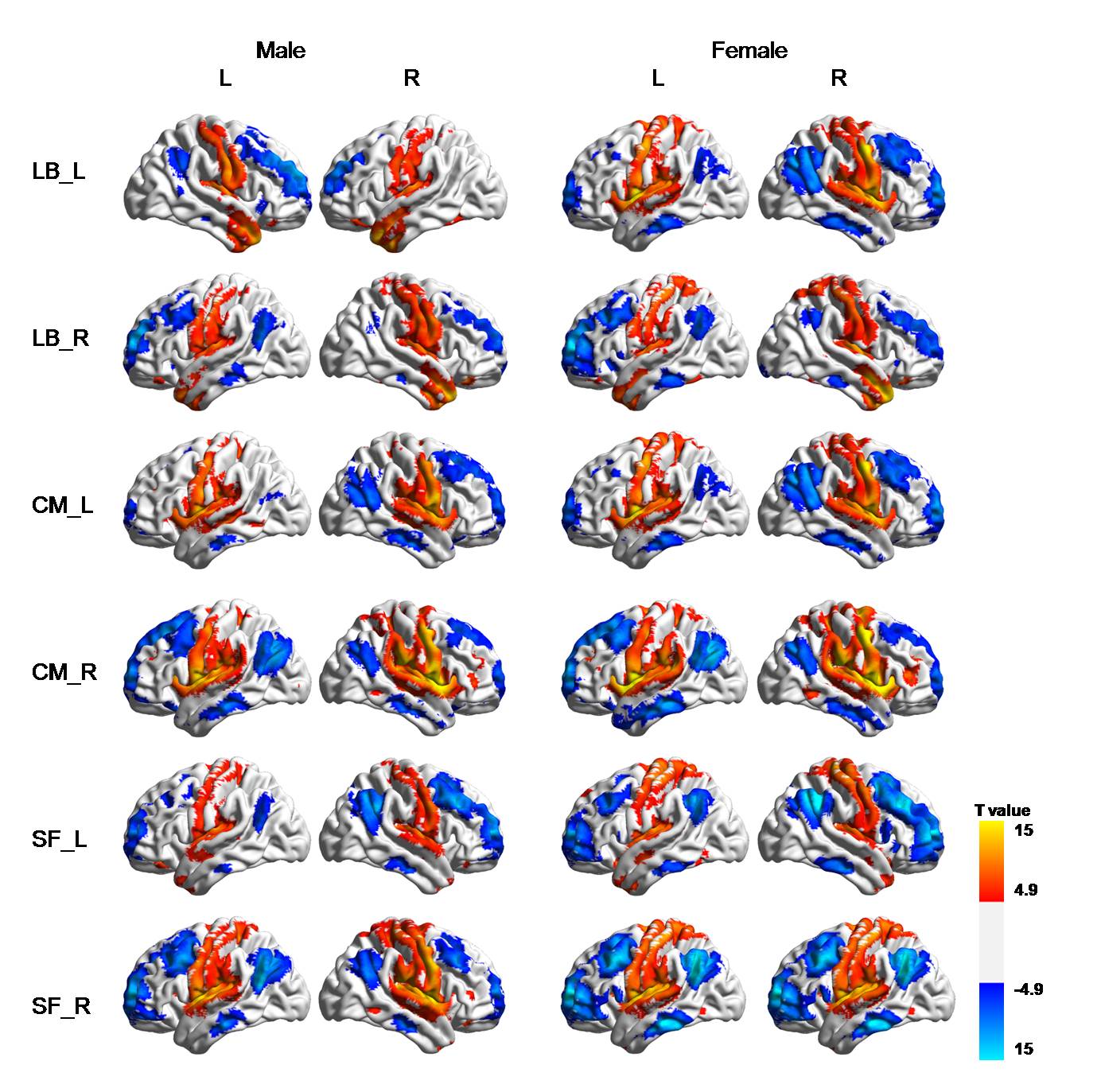


Figure S2. The patterns of RSFC for each amygdala subregion in the male and female participants, separately. Warm colors denote positive RSFC values, while cold colors denote negative RSFC values. Abbreviations: CM, centromedial subregion; RSFC, resting-state functional connectivity; L, left; LB, laterobasal subregion; R, right; and SF, superficial subregion.

**Table S1. Sex effects based on left and right SF**

| **Brain regions** | **Cluster size** | **MNI coordinates** | **Peak F-value** |
| --- | --- | --- | --- |
| L SF |  |  |  |
| L IFG/MFG | 29 | -34 40 -12 | 5.63 |
| R IFG | 16 | 50 44 6 | 5.13 |
| R IPL (SupraMarginal) | 9 | 60 -38 34 | 5.12 |
| R SF |  |  |  |
| R IFG/STG | 71 | 56 10 -2 | 5.48 |
| L IFG | 25 | -44 44 8 | 5.15 |
| L Calcarine | 8 | 0 -94 6 | 5.29 |

Abbreviations: SF, superficial subregion; L, left; R, right; IFG: inferior frontal gyrus; MFG: middle frontal gyrus; IPL: inferior parietal lobe; and STG: superior temporal gyrus.
